# Supplementary material for: Discovery of a potent, Kv7.3-selective potassium channel opener from a Polynesian traditional botanical anticonvulsant
Source: Commun Chem. 2024 Oct 10;7:233. doi: 10.1038/s42004-024-01318-9 (PMC11467302; doi:10.1038/s42004-024-01318-9)
Supplement: Supplementary file 1 — Reporting Summary [file 42004_2024_1318_MOESM1_ESM.pdf]

nature portfolio

Corresponding author(s): Geoffrey W. Abbott, PhD

Last updated by author(s): Sep 23, 2024

Reporting Summary

Nature Portfolio wishes to improve the reproducibility of the work that we publish. This form provides structure for consistency and transparency in reporting. For further information on Nature Portfolio policies, see our Editorial Policies and the Editorial Policy Checklist.

Statistics

For all statistical analyses, confirm that the following items are present in the figure legend, table legend, main text, or Methods section.

n/a	Confirmed	
☐	☒	The exact sample size (*n*) for each experimental group/condition, given as a discrete number and unit of measurement
☐	☒	A statement on whether measurements were taken from distinct samples or whether the same sample was measured repeatedly
☐	☒	The statistical test(s) used AND whether they are one- or two-sided<br>*Only common tests should be described solely by name; describe more complex techniques in the Methods section.*
☒	☐	A description of all covariates tested
☐	☒	A description of any assumptions or corrections, such as tests of normality and adjustment for multiple comparisons
☒	☐	A full description of the statistical parameters including central tendency (e.g. means) or other basic estimates (e.g. regression coefficient) AND variation (e.g. standard deviation) or associated estimates of uncertainty (e.g. confidence intervals)
☐	☒	For null hypothesis testing, the test statistic (e.g. *F*, *t*, *r*) with confidence intervals, effect sizes, degrees of freedom and *P* value noted<br>*Give P values as exact values whenever suitable.*
☒	☐	For Bayesian analysis, information on the choice of priors and Markov chain Monte Carlo settings
☒	☐	For hierarchical and complex designs, identification of the appropriate level for tests and full reporting of outcomes
☒	☐	Estimates of effect sizes (e.g. Cohen's *d*, Pearson's *r*), indicating how they were calculated

Our web collection on statistics for biologists contains articles on many of the points above.

Software and code

Policy information about availability of computer code

Data collection	pClamp10 software (Molecular Devices, Sunnyvale, CA, USA)
Data analysis	pClamp10 software (Molecular Devices, Sunnyvale, CA, USA) ; Graphpad Prism software (GraphPad, San Diego, CA, USA) ; Excel (Microsoft) ; UCSF Chimera (https://www.rbvi.ucsf.edu/chimera); Jmol, http://jmol.org/

For manuscripts utilizing custom algorithms or software that are central to the research but not yet described in published literature, software must be made available to editors and reviewers. We strongly encourage code deposition in a community repository (e.g. GitHub). See the Nature Portfolio guidelines for submitting code & software for further information.

Data

Policy information about availability of data

All manuscripts must include a data availability statement. This statement should provide the following information, where applicable:

- Accession codes, unique identifiers, or web links for publicly available datasets
- A description of any restrictions on data availability
- For clinical datasets or third party data, please ensure that the statement adheres to our policy

Source data for Figures 1-8 are available at Dryad data repository: https://doi.org/10.5061/dryad.547d7wmhn

Human research participants

Policy information about studies involving human research participants and Sex and Gender in Research.

Reporting on sex and gender	no human research
Population characteristics	no human research
Recruitment	no human research
Ethics oversight	no human research

Note that full information on the approval of the study protocol must also be provided in the manuscript.

Field-specific reporting

Please select the one below that is the best fit for your research. If you are not sure, read the appropriate sections before making your selection.

☒ Life sciences ☐ Behavioural & social sciences ☐ Ecological, evolutionary & environmental sciences

For a reference copy of the document with all sections, see nature.com/documents/nr-reporting-summary-flat.pdf

Life sciences study design

All studies must disclose on these points even when the disclosure is negative.

Sample size	Sample sizes for electrophysiology and seizures assays were based on previous analogous studies by us and others, e.g., PMID: 29748663; PMID: 31311306
Data exclusions	no data were excluded.
Replication	Other than screening (Figure 1), all electrophysiology studies were conducted on at least two different batches of oocytes. Screening results were performed in triplicate (technical replicates) and the top hits were subsequently validated using oocytes by different investigators to those who conducted the screening. All attempts at replication were successful.
Randomization	ocytes are sourced commerically and arrive in batches of 100. They are then randomly distributed into different groups for cRNA injection.
Blinding	Blinding was conducted for the seizure studies to avoid timing bias.

Reporting for specific materials, systems and methods

We require information from authors about some types of materials, experimental systems and methods used in many studies. Here, indicate whether each material, system or method listed is relevant to your study. If you are not sure if a list item applies to your research, read the appropriate section before selecting a response.

Materials & experimental systems

n/a	Involved in the study	
☒	☐	Antibodies
☐	☒	Eukaryotic cell lines
☒	☐	Palaeontology and archaeology
☐	☒	Animals and other organisms
☒	☐	Clinical data
☒	☐	Dual use research of concern

Methods

n/a	Involved in the study	
☒	☐	ChIP-seq
☒	☐	Flow cytometry
☒	☐	MRI-based neuroimaging

Eukaryotic cell lines

Policy information about cell lines and Sex and Gender in Research

Cell line source(s)	strain source, ATCC Manassas, VA; sub-strain source, ChanTest Corporation, Cleveland, OH, US
Authentication	Authentication was performed at Charles River, who also conducted the screening in-house.

Mycoplasma contamination	lines tested negative for mycoplasma
Commonly misidentified lines (See ICLAC register)	n/a

Animals and other research organisms

Policy information about studies involving animals; ARRIVE guidelines recommended for reporting animal research, and Sex and Gender in Research

Laboratory animals	Male C57Bl/6 mice at 2 months of age.
Wild animals	The study did not involve wild animals.
Reporting on sex	Seziures studies were conducted on male mice only.
Field-collected samples	The study did not involve animals collected in the field.
Ethics oversight	The UCI IACUC approved the seizure studies. The oocytes were from a commercial source and therefore did not require vertebrate animal approval at UCI.

Note that full information on the approval of the study protocol must also be provided in the manuscript.
